# ‘Captivating voices’: evaluation of a patient-centred animated video on excessive physical exercise and eating disorders

**DOI:** 10.1136/medhum-2024-013003

**Published:** 2025-01-20

**Authors:** Gerrit Brandt, Heike Bartel, Georgios Paslakis

**Affiliations:** 1 University Clinic for Psychosomatic Medicine and Psychotherapy, Medical Faculty, Campus East-Westphalia, Ruhr-Universitat Bochum, Luebbecke, Germany; 2 School of Cultures, Languages and Area Studies, University of Nottingham, Nottingham, UK

**Keywords:** narrative medicine, art and medicine, Film, Mental health care

## Abstract

This project aimed to evaluate the acceptance of a short, animated video addressing excessive exercise within the context of eating disorder (ED) behaviours among diverse target groups, assess its impact and explore potential associations with disordered eating risk. An online survey was conducted, recruiting 170 participants who were shown a 3-minute and 11-second long animated video portraying narratives of individuals with lived experiences related to excessive exercise and ED. Participants provided demographic information, engaged in the video evaluation answering a 9-item questionnaire and completed a subsequent ED screening and a drive for muscularity questionnaire. In an optional open-ended comment section, participants provided suggestions, feelings, ideas and criticism. Individuals identified as at risk for disordered eating reported a significantly higher personal impact of the video, including the motivation to self-reflect on their personal exercise habits. Qualitative analyses revealed themes related to suggestions for the video’s use, general reflections on sports behaviours and ED, and reactions to the video’s artistic design. This interdisciplinary project underscores the potential of artistic animated short videos co-designed with individuals with lived experience in conveying narratives and fostering introspection among individuals at risk for ED and excessive exercise behaviours. Further exploration and refinement of interdisciplinary artistic approaches are recommended to enhance effectiveness and inclusivity in addressing ED and associated behaviours.

## Introduction

 Eating disorders (EDs) are a public health concern and have far-reaching consequences for the affected individuals. Anorexia nervosa (AN), bulimia nervosa (BN) and binge eating disorder (BED) are estimated to affect approximately 8.4% of women and 2.2% of men over the course of their lives (Galmiche *et al* 2019); in addition, subclinical or atypical ED symptoms also pose an enormous challenge to global healthcare systems (Santomauro *et al* 2021). ED may lead to significant psychological and physical sequelae, especially if treatment is delayed (Micali *et al* 2015). Therefore, it is important to reach affected individuals appropriately and early in the course of the disease to reduce risk factors, promote illness awareness and encourage help-seeking, regardless of age, gender or socioeconomic status (Allen *et al* 2023; Halbeisen *et al* 2022).

The prevalence of muscle-oriented disordered eating behaviour and excessive exercise signifies a growing concern within the spectrum of ED, particularly impacting the demographic of young men (Downs and Mycock 2022), underscoring a complex intersection between body image ideals, excessive exercise and disordered eating patterns. In particular, the compulsive nature of exercise in ED has been associated with more severe ED-related psychopathology and a lower quality of life (Meneguzzo *et al* 2022).

While the focus of attention historically has centred on AN, BN and BED, the emergent prominence of muscle-related body image concerns, especially in men, necessitates a deeper understanding of how to reach potentially affected and/or at-risk individuals more adequately. As this issue gains visibility, it emphasises the urgent need for increased awareness, interdisciplinary research and tailored public health strategies to address the multifaceted dimensions of ED, encompassing excessive exercise and its impact on mental health and well-being.

In this context, online prevention and educational programmes may serve as a crucial platform for reaching at-risk individuals, particularly those of younger age. This is particularly true in light of the fact that social media consumption in general and the consumption of short videos in particular have increased dramatically in recent years (Meltwater 2016). Short videos are typically 10–180 s long and can be distributed on social media (eg, Instagram, TikTok) or YouTube via messenger services (eg, WhatsApp, Signal) or email. An inherent challenge with this form of dissemination is the heterogeneity of the target groups. In addition to those affected or at risk, target groups may also include professionals—within and outside the healthcare system—playing an essential part in the prevention and treatment of ED, such as primary care physicians, medical students, carers, school staff, fitness instructors in sports clubs or gyms and others.

### Study aim

The aim of this study was to conduct an exploratory online survey to evaluate the impact of a short, animated video addressing excessive exercise in the context of ED-related behaviours across different target groups within the general public. Special attention was given to gender groups, with further focus on individuals at risk for EDs and counsellors/practitioners. Using a mixed-method approach that combined quantitative and qualitative analyses, the study aimed to comprehensively assess the video’s effects across the various target groups, offering nuanced insights into its reception and impact.

## Method

### Study design

The survey took place online between August and September 2023. Participants were recruited using a convenience sampling approach. Clinical staff members were contacted via medical staff mailing lists from the healthcare network of Mühlenkreiskliniken in North Rhine-Westphalia, Germany. To effectively target at-risk groups, we also used mailing lists from the Ruhr-University Bochum, which addressed students from various academic disciplines. To broaden the reach and enhance diversity, participants were encouraged to share the survey within their networks, thus employing a snowball sampling method. Participants were neither provided with incentives for their participation, nor were any incentives offered as a prospect. All participants provided their explicit consent to take part in the study, on which they were shown a brief animated video. No information about the development and production of the video was provided to participants. Participants were asked to provide demographic information (among others, whether they engaged in physical exercise in professional or leisure contexts or not, as well as if they were involved in the professional care or other forms of support for individuals with disordered eating behaviours), followed by questions to rate the video. The next option was to either conclude participation in the study or complete two additional questionnaires. Overall, n=259 individuals participated in the study, with 89 (34.36%) excluded due to missing data (n=84) or unusually short processing times (<200 s; n=5), to ensure the dataset’s integrity. In total, n=170 completed the video evaluation, while n=147 of these also filled out the two optional subsequent questionnaires. Also, n=55 participants provided additional free-text comments. Table 1 displays the participant sociodemographic characteristics for each of the specified survey groups. There were no differences between the groups regarding age and gender distribution (p=0.65 and p=0.80, respectively).

**Table 1:** Participant sociodemographic characteristics per survey group

	Video evaluation	Video evaluation+questionnaires	Open-ended feedback
n	170	147	55
Age, M (SD)	36.31 (13.61)	35.9 (13.39)	37.56 (13.30)
Women, n (%)	112 (65.9%)	100 (68.0%)	34 (68.0%)
Men, n (%)	52 (30.6%)	42 (28.6%)	17 (30.9%)
Diverse, n (%)	6 (3.5%)	5 (3.4%)	4 (7.3%)

### Video design

The presented video was developed in English at the University of Nottingham and translated into German in close collaboration with the Ruhr-University Bochum. The 3-minute and 11-second long animated video provided insights into the relationship between excessive exercise and disordered eating from the perspectives of three individuals (two women and one man) who engage in excessive exercise as part of AN, BN and muscularity-oriented ED.^1^ The creation of the video involved the active participation of individuals with lived experience, with their experience authentically depicted verbatim in the video. Furthermore, experts in animation and video music were engaged and contributed their expertise to the project (see the Acknowledgements section). The video was conceived as a fusion of artistic and humanities-based concepts—specifically, the cinematic animated interpretation of narratives based on lived experiences—and medical objectives. It was grounded on the interdisciplinary research approaches of Health Humanities and applying principles based on Narrative Medicine (Charon 2006).

### Video evaluation

In a collaborative, multiprofessional process, the authors developed the 9-item evaluation questionnaire. It assessed (1) the overall liking of the video (appeal) and included questions regarding (2) perceived general relevance, (3) impact on personal awareness, (4) impact on knowledge about excessive exercise, (5) increase in motivation/curiosity to learn more about the topic, (6) self-reflection on one’s own training behaviour, as well as (7) emotional engagement, (8) personal significance of the video, and finally, (9) the willingness to recommend the video to others. The open-ended comment section encouraged participants to provide suggestions, feelings, ideas and criticism.

### Screening for eating disorders and drive for muscularity

For the optional questionnaire survey, we used the German short version of the Eating Attitudes Test (EAT-8; Richter *et al* 2016). The EAT-8 consists of eight yes-or-no questions to assess core symptoms associated with ED, with a total score of ≥2 considered a conservative cut-off score. Additionally, the German version of the Drive for Muscularity Scale (DMS; Waldorf *et al* 2014) was used. This 15-item questionnaire measures muscle-related cognitions, as well as muscle-related behaviour and has a high internal consistency (Cronbach’s α=0.9).

### Data aggregation and analysis

The overall results for the EAT-8 and DMS were aggregated according to the specifications of each questionnaire. For group comparisons between individuals with and without a high risk of disordered eating, a cut-off ≥2 in the EAT-8 was adopted. Descriptive results are presented as means, relative frequencies and standard deviations. The significance level for all analyses was set at p≤0.05. Due to the small sample size, the group of individuals who identified as gender diverse (n=6) was not included in the final statistical analyses.

Since the data from EAT-8 and DMS were not normally distributed, non-parametric tests such as the Mann-Whitney U test were conducted. Additionally, bivariate correlation models were computed to analyse inferences between the variables. The statistical analysis was conducted using SPSS Statistics V.30 for Windows (IBM Corp 2023). The qualitative analysis of the open-ended comments (n=55) was conducted using reflexive thematic analysis following Braun and Clarke’s (2006) approach, which generated three themes through their 6-step method.

### Patient and public involvement statement

In this research project, we acknowledge that persons with lived experience were not directly involved in the design, conduct, reporting or dissemination plans of our study. However, it is important to note that affected individuals directly contributed to the video material used and thus had a certain degree of involvement.

## Results

Of the total 170 participants, 65.9% identified as women, 30.6% as men and 3.5% as gender diverse. The average age across all respondents was 36.31 (13.61) years, with men averaging 33.35 (11.8) years, younger on average than women at 37.66 (14.15) years, although this difference was not statistically significant (p=0.271).

Of those respondents, 84.1% engaged in physical exercise/sports (including seven individuals in a professional context), and 15.9% reported not participating in any sports activities. Furthermore, 17.1% of respondents stated their involvement in counselling or treatment of individuals with disordered eating behaviours and/or excessive exercise behaviours. Notably, the group offering counselling and treatment, with an average age of 44.48 years (SD=12.31), was significantly older than the rest of the sample (p<0.001).

### Quantitative analysis

Table 2 summarises the results of gender group comparisons, indicating that women were more likely to recommend the video to others (p=0.038). Women had higher median scores in the DMS questionnaire concerning the total score (p=0.038) and the muscle-related cognitions (MC) subscale (p=0.030). Regarding the remaining questions and the EAT-8 score, values between men and women remained comparable (ps≥0.133).

**Table 2:** Group comparisons by gender

Variable	Men			Women			P value	
	n	Md	%pos	n	Md	%pos		
Appeal	52	1.00	90.4	112	1.00	89.3	0.779	
Relevance	52	1.00	94.2	112	1.00	97.3	0.356	
Awareness	52	1.00	61.5	112	1.00	62.5	1.000	
Knowledge	52	1.00	51.9	112	0.00	42.9	0.465	
Curiosity	52	0.00	36.5	112	0.00	37.5	0.709	
Self-reflection	52	0.50	50.0	112	0.00	49.1	1.000	
Emotion	52	0.00	36.5	112	1.00	53.6	0.069	
Personal	52	0.00	17.3	112	0.00	20.5	1.000	
Recommend	52	1.00	80.8	112	1.00	92.0	**0.038**	
	**n**	**Md**	**IQR**	**n**	**Md**	**IQR**	**U**	**P value**
EAT-8 total	42	2.50	4.0	103	2.0	4.0	2046.5	0.608
DMS total	42	4.60	1.28	103	5.13	0.93	1635.5	**0.038**
DMS MC	42	3.71	1.61	103	4.86	0.96	1614.0	**0.030**
DMS MB	42	5.29	1.50	103	5.43	1.25	1765.5	0.133

The Mann-Whitney U test was applied for comparisons involving the EAT-8 and DMS scores. Fisher’s exact test was used to analyse the evaluation question. Values in bold indicate statistical significance.

DMS MB, muscle-related behaviour, subscale of the Drive for Muscularity Scale; DMS MC, muscle-related cognitions, subscale of the Drive for Muscularity Scale; DMS total, Drive for Muscularity Scale total score; EAT-8 total, Eating Attitude Test-8 total score; Md, Median.

Table 3 shows the comparison between participants screened as being at risk and those without a risk for disordered eating behaviours or ED according to the EAT-8, where individuals at risk (ED+) more frequently reported that the video prompted them to reflect on their own training behaviours (p=0.024; item ‘Self-Reflection’). Furthermore, participants at risk more commonly stated that the video personally affected them (p<0.001; item ‘Personal’). Additionally, individuals at risk had significantly higher scores in the global score of the DMS and in both subscales (ps<0.001), respectively. All other items remained comparable (ps>0.402).

**Table 3:** Group comparisons by eating disorder screening

Variable	ES^-^			ES^+^			P value	
	n	Md	%pos	n	Md	%pos		
Appeal	77	1.00	89.6%	73	1.00	86.3%	0.619	
Relevance	77	1.00	96.1%	73	1.00	95.9%	1.00	
Awareness	77	1.00	58.4%	73	1.00	65.8%	0.402	
Knowledge	77	0.00	46.8%	73	0.00	43.8%	0.745	
Curiosity	77	0.00	35.1%	73	0.00	42.5%	0.403	
Self-reflection	77	0.00	41.6%	73	1.00	60.3%	**0.024**	
Emotion	77	0.00	49.4%	73	1.00	50.7%	1.00	
Personal	77	0.00	3.9%	73	0.00	41.1%	**<**0.001	
Recommend	77	1.00	89.6%	73	1.00	84.9%	0.465	
	**n**	**Md**	**IQR**	**n**	**Md**	**IQR**	**U**	**P value**
DMS total	77	4.67	1.32	73	5.33	0.80	1380.5	**<0.001**
DMS MC	77	4.36	1.68	73	5.00	1.00	1537.5	**<0.001**
DMS MB	77	5.00	1.86	73	5.71	0.57	1494.5	**<0.001**

The Mann-Whitney U test was applied for comparisons involving the EAT-8 and DMS scores. Fisher’s exact test was used to analyse the evaluation question. Values in bold indicate statistical significance.

DMS MC, muscle-related cognitions, subscale of the Drive for Muscularity Scale; DMS MS, muscle-related behaviour, subscale of the Drive for Muscularity Scale; DMS total, Drive for Muscularity Scale total score; ED, eating disorder; ES-, Eating Attitude Test-8 total score ≤ 2; ES+, Eating Attitude Test-8 total score > 2; Md, Median.

Table 4 summarises the results of those involved and not involved in counselling or treatment. Those involved in counselling or treatment reported less frequently that the video raised their awareness (p=0.021), knowledge (p=0.013) or self-reflection (p=0.041). They also less frequently reported that the video personally affected them (p=0.011). Individuals in counselling or treatment are more likely to be familiar with the issue of excessive exercise in the context of ED behaviours, thus already possessing greater awareness, knowledge and self-reflection, and, as a result, are less likely to be personally affected by the content. This was also mirrored in lower scores on the EAT-8 (p<0.001) and the DMS MB (muscle-related behaviours subscale; p=0.05). At the same time, both those involved and not involved in counselling or treatment rated the appeal and relevance of the video as being very high (93.1% and 100% positive answers, respectively); 98.7% of those involved in counselling or treatment would recommend the video. These high scores were not statistically different from those in the rest of the sample (ps>0.533).

**Table 4:** Group comparisons by involvement in counselling or treatment

Variable	Involved in counselling or treatment			Not involved in counselling or treatment			P value	
	n	Md	%pos	n	Md	%pos		
Appeal	29	1.00	93.1%	141	1.00	87.2%	0.533	
Relevance	29	1.00	100.0%	141	1.00	95.0%	0.604	
Awareness	29	0.00	41.4%	141	1.00	65.2%	**0.021**	
Knowledge	29	0.00	24.1%	141	1.00	50.4%	**0.013**	
Curiosity	29	0.00	44.8%	141	0.00	35.5%	0.400	
Self-reflection	29	0.00	31.0%	141	1.00	53.2%	**0.041**	
Emotion	29	1.00	51.7%	141	0.00	46.8%	0.686	
Personal	29	0.00	3.4%	141	0.00	23.4%	**0.011**	
Recommend	29	1.00	89.7%	141	1.00	87.2%	1.00	
	**n**	**Md**	**IQR**	**n**	**Md**	**IQR**	**U**	**P value**
EAT-8 total	29	1.00	3.00	141	3.00	5.00	943.5	**<0.001**
DMS total	29	5.40	0.80	141	5.00	1.30	1957.0	0.051
DMS MC	29	4.93	0.86	141	4.71	1.57	1852.5	0.155
DMS MB	29	5.71	0.75	141	5.43	1.43	1958.0	**0.05**

The Mann-Whitney U test was applied for comparisons involving the EAT-8 and DMS scores. Fisher’s exact test was used to analyse the evaluation question. Values in bold indicate statistical significance.

DMS MB, muscle-related behaviour, subscale of the Drive for Muscularity Scale; DMS MC, muscle-related cognitions, subscale of the Drive for Muscularity Scale; DMS total, Drive for Muscularity Scale total score; EAT-8 total, Eating Attitude Test-8 total score; Md, Median.

A multiple linear regression revealed that scores in the EAT-8 predicted reflection on one’s own training behaviours, even when controlling for gender, β=0.25, p=0.005; however, the DMS scores did not predict reflection on one’s own training behaviours, β=−0.125, p=0.168, when controlling for gender (table 5). Furthermore, our predictive analyses indicated that both the EAT-8 and DMS total scores predicted personal involvement, β=0.428, p<0.001, and β=−0.380, p<0.001, even when controlling for gender (table 5).

**Table 5:** Regression analyses using (1) reflection on training behaviours, and (2) personal involvement as criterion

Criterion: Reflection on training behaviours					
	B	SE	Beta	t	Sig.
(Constant)	0.712	0.293		2.430	0.016
EAT-8 total	0.049	0.017	0.253	2.836	0.005
DMS total	−0.073	0.053	−0.125	−1.386	0.168
Gender	−0.016	0.089	−0.015	−0.181	0.857

Criterion: Personal Involvement					
	B	SE	Beta	t	Sig.
(Constant)	0.908	0.186		4.883	<0.001
EAT-8 total	0.068	0.011	0.428	6.236	<0.001
DMS total	−0.184	0.034	−0.380	−5.471	<0.001
Gender	−0.045	0.057	−0.049	−0.793	0.429

Note: Gender variable was coded as a dummy variable where Man=1 and Woman=0 (reference category).

DMS, Drive for Muscularity Scale; EAT-8 total, Eating Attitude Test-8 total score.

### Qualitative analysis

Part of the evaluation involved an open-ended comment section that prompted participants to express comments, feelings, ideas, criticism (eg, ‘what was missing in the video?’, ‘what would you do differently?’, ‘what did you particularly like?’) in German (later translated into English). The open-ended comments had an average word count of 36.5 (SD=34.78), with a range of 3 to 192 words. The free-text responses were categorised into four categories and then subjected to thematic analysis (Braun and Clarke 2006) resulting in three themes.

#### Categories

The four categories of the 55 open-ended comments were as follows: (1) consistently positive evaluation of the video (n=15); (2) consistently negative or critical evaluation (n=9); (3) mixed positive and critical evaluation (n=30); (4) neutral description (n=1).

#### Themes

The thematic analysis generated three themes: (1) suggestions for the use of the video; (2) reflections on the topics of sports behaviour and ED; (3) reactions to the artistic design of the video.

##### Suggestions for using the video

A total of 15 open-answer comments from the ‘positive’ and ‘mixed’ categories provided suggestions for the professional use of the video. Among these, seven comments noted that the video conveyed basic information on the topic of ED and excessive training, lacking explicit expertise for ‘professionals’, but deeming it suitable for ‘newbies’. Specifically, for newcomers in relevant professional fields newly engaging with the topic, it served as a ‘good introduction’, described as ‘fantastic for a first overview’, providing a ‘comprehensive package for problem explanation and guidance’, although being ‘more suitable for laypeople’ and needing ‘more specifics and facts’. Potential target groups and uses for the video mentioned included: ‘trainers and fitness studios’; ‘affected individuals/family members’ and ‘patients’; ‘schools’; professional ‘training programmes’ and the ‘general practitioner context’. Three comments articulated that the video prompted them to reflect on their own sports behaviour.

Nine comments from the ‘positive’, ‘critical’ and ‘mixed’ categories pointed out gaps and made suggestions for further work on the theme for professional use: additional and more detailed information about counselling centres; additional ‘info boxes’ in the video; greater focus on psychological backgrounds and consequences for the problems depicted in the video, and a more complex presentation of their possible solutions. Two commented: ‘clear message, well conveyed’ and ‘Nothing was missing’.

##### Reflections on the topics of sports behaviour and eating disorders

Six responses from the categories ‘positive’, ‘mixed’ and ‘critical’ offered comments regarding sports in connection with excessive training and ED behaviours. Two suggested to include additional testimonials to the video that should also show the ‘positive aspects of sports’, such as ‘health, meeting friends, illness prevention, etc.’. One comment reported that the video led to criticism of a viewpoint that ‘society generally views sports as something inherently good’. One comment emphasised that the topic of ‘sports behaviour and eating disorders’ should be depicted in a much more complex manner in the video; another raised the open question of when training becomes excessive, and another requested more extensive reflection on the ‘why’ in seeking approval in sports.

Six of the 55 comments were positive toward the diversity depicted in the video, presenting excessive training behaviour and disordered eating as an issue affecting ‘diverse groups’: ‘both women and men’, ‘not just underweight girls or women’, ‘underweight and overweight individuals’, ‘not only thin or very athletic-looking individuals but everyone’. One comment identified the following ‘stereotype’: “In the end, the woman does yoga, the man boxes”.

##### Reactions to the artistic design of the video

Twenty-two out of 55 comments (40%) from the open-answer comment field provided feedback on the animated video, focusing on (1) the artistic design of the video; (2) the composition and length of the video; (3) the emotional impact of the video.

##### Artistic design

There were eight positive general comments about the video: ‘beautiful presentation/design’, ‘well done’, ‘nicely done’, ‘appealing’, ‘well executed’, ‘great video’, good ‘quality of the video’, ‘great’. There were two negative comments: ‘outdated’, ‘too artificial and abstract’. Four comments addressed the portrayal of characters in the film as animated figures. One comment found the ‘comic-style’ portrayal very good compared with a real video representation, another found the ‘characters well made’. Two would have preferred the portrayal of ‘real people’.

##### Composition and length

Three comments associated the video’s brevity (3 min 11 s) with positive evaluation: ‘nicely short, touching, and concise’; ‘good and concise’; ‘short […] packed in many important aspects’. There were no negative comments about the length of the video. Two negative comments criticised the alternating film cuts between the three characters as ‘confusing jumps’ and ‘confusing’.

##### Emotional Impact

In total, six open-ended comments across the ‘positive’, ‘mixed’ and ‘critical/negative’ categories specifically focused on the emotional influence of the video. Four comments explicitly discussed the voices in the video: ‘particularly the speakers’ voices […] emotionally moved me, in connection with the stories told’; ‘pleasant narration’; ‘captivating voices’, and ‘I find the tone of the narration somewhat sentimental’. One participant would have found the portrayal of ‘real people’ instead of illustrated characters ‘more emotionally gripping’. One comment perceived the ‘mood extremely subdued; it made me sad and in a bad mood’, and a participant expressed extremely derogatory emotions toward the characters in the film, referring to them as ‘shitty bodies’ (in German: ‘Dreckskörper’).

## Discussion

The aim of this study was to evaluate the suitability of a short, animated video on excessive exercise in the context of ED behaviour with different target groups. Gender groups were a key focus, with special consideration given to individuals vulnerable to EDs and individuals involved in counselling or treatment. Our findings revealed that individuals identified at risk for ED showed a higher tendency to engage in introspection concerning their personal exercise habits. Moreover, these at-risk participants more frequently acknowledged a personal impact from the video. These outcomes collectively emphasise the video’s distinct influence on individuals who may be predisposed to ED.

The animated film was designed to articulate the individual experiences of three individuals with ED in connection with excessive exercise, narrated in their own words and as part of a collaborative creative process they were involved in. The primary focal points included: (1) showcasing diversity in the portrayal of ED and those affected by them; (2) avoiding the perpetuation of stereotypes surrounding ED; and (3) addressing muscle-oriented ED in addition to the diagnostic criteria of the Diagnostic and Statistical Manual of Mental Disorders for ED (DSM; APA 2013).

Excessive exercise is a relevant issue in EDs, affecting up to 80% of patients with the restrictive type of anorexia nervosa (Hebebrand *et al* 2003; Keyes *et al* 2015). It is argued that it is not the amount of exercise rather than its objective to regulate weight and shape the body, as well as the compulsive character of exercise that defines it as being excessive (Cook and Hausenblas 2008). Indeed, the compulsive aspect of exercise is a better predictor of disordered eating than the quantity of exercise itself (Holland *et al* 2014). Excessive exercise has been associated with the severity of the ED-specific psychopathology, longer hospitalisations and a poorer outcome, including shorter times to relapse and a chronic course of the ED (Bratland-Sanda *et al* 2010; Peñas-Lledó *et al* 2002).

Our quantitative analysis revealed that women were more likely to recommend the video to others. Furthermore, individuals at risk for EDs more frequently reported that the video prompted them to reflect on their own training behaviours and that they were more personally affected by its content. Predictive analyses showed that EAT-8 scores predicted reflection, while both DMS and EAT-8 scores predicted personal involvement with the video. This suggests that the video has had a positive impact, particularly among individuals with a higher ED risk. Additionally, we observed an overall positive evaluation from individuals involved in counselling or treatment, particularly regarding the video’s appeal and the likelihood of recommending it to others.

The qualitative content analysis yielded, that participants acknowledged and appreciated the diversity within the video concerning gender representation and its inclusive portrayal of various body types. However, a remark on the gender-specific representation of sports— yoga being female and boxing male—highlighted the demand within research and creative industries for continuous reflection on unintentional perpetuation of stereotypes. Furthermore, pragmatic conclusions for the application and expansion of the video emerged: (1) inclusion of additional customised data on sport and ED; (2) inclusion of support information, including referrals to counselling services and detailed information on sport-related issues.

Of those participants, 58% made explicitly positive comments about the animated video when asked for open-ended feedback and three expressively praised its brevity (3 min and 11 s). Research has drawn parallels between a video’s brevity and its effectiveness in professional training (Knapp *et al* 2022). The duration, which is usually between 10 and 180 s, is crucial for the distribution of such videos on social media platforms such as YouTube or via messenger services (eg, WhatsApp, Signal). The latter represents an important dissemination medium (Meltwater 2016), especially for people of a younger age, and has the potential to reach broad target groups. However, despite the need for brevity, feedback gathered in this study indicated the need for clarity and the accessibility of composition.

Creative filmmaking and the conveyed narratives of lived experiences are significant contributors to enhancing empathy, understanding, self-reflection and engagement in interventions addressing ED in the context of excessive exercise. These elements play a crucial role in fostering relatability, capturing attention and facilitating emotional connections, thereby enabling a more profound impact on individuals grappling with these issues. Comments from our qualitative analysis indicated that the video offered more than factual knowledge, generating an emotional impact predominantly perceived as positive among participants. However, comments also suggested providing more facts and knowledge to affected individuals and implementing relevant trigger warnings. Regarding the emotional impact of the video, participants particularly emphasised the effect of voices in the animation. Bolaki (2016) discussed the use of voice in animated films concerning mental health issues and the evocative characteristics of voice, such as intonation, breathing frequency, and the connection or lack thereof between a cinematic voice and the visual representation (eg, ‘off-screen’ or ‘acousmatic’). Additionally, the positive or critical evaluation of illustrated characters rather than real people, offering identification opportunities in some comments, could also prompt discussions aligning with research opinions. McCloud and Martin (1993) argued that potentially everyone can recognise themselves in the simplified depiction of an animation, but very few in a realistic portrayal, such as in a photo or film: “we don’t just observe the cartoon, we become it”.

Doctors, nurses, therapists and other medical professionals can only be as good as the training they receive; Morgan (2008) summarised this in an evaluation of educational and knowledge gaps in the field of ED, particularly concerning under-represented groups. Delderfield (2018) emphasised the necessity for approaches that incorporate individual experiences, citing deficiencies in research and training concerning ED in men, boys and individuals from sexual and gender minorities (see also Halbeisen *et al* 2022). This applies not only to these under-researched groups but also to the investigation of AN and BN, where current therapies often display low recovery rates (Quadflieg and Fichter 2019; Eddy *et al* 2017; Sala *et al* 2023). Recent research approaches underscore the need for a shift in perspective and closer collaboration with those affected in researching and treating ED (Bryant *et al* 2022; Kenny and Lewis 2021). Brinchmann *et al* (2022) call for a change in ED research, moving away from the dominance of prevailing ED narratives in the language of psychiatric diagnostics and opening up to ‘engaging with the narratives of those living with an ED with curiosity, openness, and willingness to learn’. Conti *et al* (2016) framed this as a need for ‘a different kind of listening’.

Health Humanities approaches have the potential to articulate and convey these new perspectives ‘outside and beyond the biomedical gaze’ (Crawford *et al* 2015). The goal is to expand empirical research, diagnostic criteria and quantitative analysis by incorporating contributions from affected individuals and integrating art and the insights and critical approaches of the humanities. In this context, the voices of those with lived experiences and their artistic representation in the medium of animation films can play a crucial role. Prominent evaluations, such as that of another animation film with a similar concept as part of a training session on men with ED (Bartel 2023), speak to the promising potential of such approaches. Not only was there a quantitative increase in knowledge among participants, but comments also indicated a changed attitude and greater openness to considering ED among previously underrepresented groups and perceiving patients differently (Bartel 2022). A nurse articulated the learning impact of this patient-oriented short film: “I am unlikely to forget these voices of men in my future practice. It made me listen more and will lead to helping more men with an eating disorder” (Bartel 2020).

### Limitations

A few limitations of this study should be acknowledged to provide context for the interpretation of the findings. In this study, no incentives were provided, which may have influenced participant engagement and contributed to a higher rate of non-completion. Future studies could consider offering incentives to enhance participation, reduce dropout rates, and improve the representativeness of the sample. The exploratory nature of the study prioritised identifying potential patterns and relationships over confirmatory analyses. While this approach aligns with the study’s goals, it may increase the risk of type I errors. Future research should address this limitation by applying appropriate corrections, such as the Bonferroni adjustment, to ensure greater statistical rigour. Furthermore, while the drive for muscularity and ED risk were in the focus of this study, other comorbid psychological issues, such as depressive symptoms or anxiety, were not assessed. Including a variety of comorbidities in future research could provide a more comprehensive understanding of the effectiveness of video-based interventions. Additionally, while gender was assessed beyond the binary man versus woman categories, the small number of individuals identifying outside the binary made a more detailed analysis impossible; this should be addressed in future studies with a larger and more diverse sample. Finally, future research should also include the assessment of sexual orientation, as it has recently been shown to be linked to differing drives toward certain body sizes and associated behaviours (Dal Brun *et al* 2024; Zaiser *et al* 2024).

## Conclusion

Interdisciplinary research and art integration have the potential to expand our understanding of ED, offering diverse perspectives from medicine, psychology and the arts. Artistic mediums, such as videos and narratives, can enable profound expression and communication, increase compassion and understanding, and promote new and different learning and listening to the voices of individuals with lived experience with particular potential to engage with under-represented groups and reach a social media-oriented generation. However, the results of this study suggest to further explore the potential of interdisciplinary artistic approaches regarding mental illnesses in general and ED in particular.

## Data Availability

Data are available upon reasonable request.

## Footnotes

The English version of the video is available at: https://www.youtube.com/watch?v=s5DgLQP8s8g, the German version of the video is available at: https://youtu.be/VqFPE-mhpZ0?si=2Va0isLXpkmgfIWO
